# Collective Traumas and the Development of Leader Values: A Currently Omitted, but Increasingly Urgent, Research Area

**DOI:** 10.3389/fpsyg.2019.01009

**Published:** 2019-05-03

**Authors:** Lara A. Tcholakian, Svetlana N. Khapova, Erik van de Loo, Roger Lehman

**Affiliations:** ^1^ Department of Management and Organization, School of Business and Economics, Vrije Universiteit Amsterdam, Amsterdam, Netherlands; ^2^ Faculty of Organisational Behavior, INSEAD Asia and Europe Campus, INSEAD, Fontainebleau, France; ^3^ Entrepreneurship and Family Enterprise, INSEAD Asia and Europe Campus, INSEAD, Fontainebleau, France

**Keywords:** transmission of collective trauma, collective trauma, leader development, leader values, leader behavior

## Abstract

The number of worldwide traumatic events is significant, yet the literature pays little attention to their implications for leader development. This article calls for a consideration of how collective trauma such as genocide and the Holocaust can shape the values of leaders, who are second- and third-generation descendants. Drawing on research on the transgenerational transmission of collective trauma and leader values, we show how collective trauma resides in (1) cultural rituals and artifacts, (2) community events and commemorations, and (3) family narratives is transmitted to leader descendants through at least three channels: social learning, social identity, and psychodynamics. We also offer propositions that recommend ways in which the transmission of these repositories can shape certain leader values that guide leader behaviors. Our conceptual review suggests that the transmission of collective trauma on leader development and leader values remains under-researched, offering prospects for new research and learning on the origins and seeds of leader development.

## Introduction

The influence of social context on leader development has been an important topic in leadership development literature. While much has been said about how family, social environment, education, and cultural background inject values and aspire leaders to behave (House et al., 2002; Shamir et al., 2005; Day et al., 2014; Mumford et al., 2015; Nguyen et al., 2018), one topic that remains persistently omitted is the effects of collective traumas on leader development. We believe that this topic is increasingly relevant, considering the great diversity of leaders that lead organizations today.

Research in psychology and cognitive psychology shows that traumas experienced by individuals in their childhood become part of who they are as adults (Danieli, 1998; Kidron, 2004; Philippe et al., 2011; Ogle et al., 2013). For example, several studies have examined how exposure to trauma experienced during childhood (including exposure to stories of collective torture and murder) reveals positive or negative consequences in individuals’ transitions to adulthood (Yehuda et al., 1998; Ogle et al., 2013).

In the leadership literature, there is also evidence that social and situational backgrounds can influence a leader (Brown and Treviño, 2006). Research has shown that leaders associated with a collective trauma carry a set of life stories that stimulate their values and convictions (Avolio and Gardner, 2005; Shamir and Eilam, 2005). These values are relevant when leaders bring them to the decision-making table. How these values emerge or how they are transmitted by collective trauma is a major way to substantiate leaders. However, to our knowledge, hardly any research exists that makes a connection between transmitted collective traumas and consequent leadership.

In this article, we aim to contribute to fill this research and conceptual gap and offer a theoretical framework regarding the consequences of collective trauma transmission for leader values. We draw on the case of the transmission of genocide as a collective trauma to develop our propositions. Several genocides have occurred over the course of history, and individuals today continue to remember or hear stories about how their collective group was subject to atrocities. These stories influence how the family or community commemorates or remembers these collective traumas, and we seek to answer how the transmission of collective trauma shapes leader values and behaviors.

**Figure 1:**
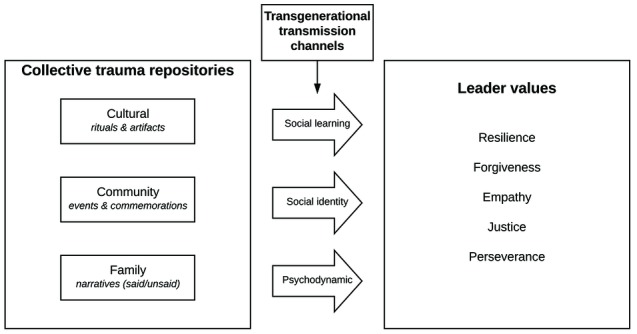
The theoretical framework on the transmission of collective trauma on leader values.

Our article makes three contributions. First, to understand the transmission process and its role in the development of leader values, we start by exploring how the memories of collective trauma reside in cultural artifacts or rituals, communities, and family narratives (Danieli, 1998; Rousseau and Drapeau, 1998). Second, from these collective repositories, we identify how collective trauma is transmitted through three channels: social learning, social identity, and psychodynamics. Social learning theory connects human behavior with the influences of the environment and role models (Bandura et al., 1961) as learned by the collective through national artifacts or rituals. The transmission process may also occur through community involvement related to commemorations or events that keep alive the memory of the collective trauma. Social identity theory creates a bridge between the collective and the members of the community through a connection to the values of the community members, i.e., through the “social categorizations” with which community members identify themselves (e.g., ethnicity and religion) (Tajfel and Turner, 1979; Turner and Mavin, 2008). Finally, the process of transmission connects the community with the family through narratives and stories. Psychodynamic theory helps us understand the conscious and unconscious dynamics behind behaviors stemming from the origin of the family and its relevant collective trauma life stories that are told or felt, during the formative years of the descendant (Zaleznik, 1963; Kets de Vries et al., 2013). Children raised in a family that has survived a collective trauma will identify themselves with the experiences, identities, and behaviors of their family’s collective trauma and glory (Volkan, 2009). Even if descendants have not physically witnessed collective atrocities such as genocide, for instance, they can still be affected by the stories or memories shared by their caregivers and social groups. Based on these identified transmission channels, we develop a model (Figure 1) that proposes how the transmission of collective trauma develops or shapes leader values and proposes five types of values as listed below. The third contribution and overall goal of this article offers propositions grounded in the three theories identified above to shed new light on the consequences of the transmission of collective trauma on the development of leader values and to help outline new directions for future research on leader development (focus on the individual) and eventually leadership development (focus on organizational leadership capacity and relations) as these theories become increasingly important in global, diverse organizations.

Our article is structured to assess primarily the constructs relevant to collective trauma and leader values. We then offer three types of collective trauma repositories that are vivid in the memory of descendants and suggest the channels through which these repositories are processed or transmitted. We finally propose a model that explains how the transmission of collective trauma repositories can shape certain leader values as proposed by our research: resilience, forgiveness, empathy, justice, and perseverance.

## Collective Trauma and Leader Values

Leader values and behaviors can lead to important organizational failures and successes. Consequently, it is important to understand the background of individual leaders to make sense of their relevant emergent behaviors as leaders. Collective trauma helps us understand the possible antecedents that can shape leader development, namely, as relevant to values. Although not all leaders have a history of collective trauma, the number of individuals who do have such a history remains significant (e.g., Native Americans; victims of the Armenian genocide; victims of the Holocaust; victims of genocides in Cambodia, Rwanda, Bosnia, and Darfur; and even more recently, victims of the Syrian civil war).

Collective trauma (or historical trauma) is an emotional and psychological stress that has affected a large group and that moves across generations (Brave Heart, 2003); it affects members of a group, who have a strong affiliation with the collective group’s identity (Mangassarian, 2016). The Jewish Holocaust is the most recognized and researched collective trauma, but there are several others: the Armenian genocide (Charny and Fromer, 1998; Kalagian Blunt, 2014), the Ukrainian genocide (Bezo and Maggi, 2015), the Cambodian genocide (Tyner et al., 2012; Nou, 2013), and the Native American genocide (Whitbeck et al., 2004; Evans-Campbell, 2008). In each case, there has been a process of collective mourning and the development of collective emotions.

Building on Eyerman’s insight into collective memory, which “unifies a group through time and space” (Eyerman, 2004, p. 161), various studies have begun to show how cultural practices such as commemorations or narratives about the past help develop emotional and cognitive imprints that are revived or recalled in the individual (Eyerman, 2004). Collective memory (or repository as the term we use in our research) refers to the individual recollections that are stored from collective representations or shared identities (from traditions, rituals, language, collective events, commemorations, schooling, and narratives) (Olick, 1999). Collective trauma, an event that may have taken place years ago, or even a century ago, may feel as though it took place only recently and then forms part of the collective identity (Volkan, 2001; Fromm, 2012).

Childhood experiences are key ingredients in the development of leaders, and one component relevant to our research relates to the leader’s collective background (Popper and Amit, 2009). Murphy and Johnson (2011), in his research on the seeds of leader development, focus on early developmental factors that identify the roots of leadership skills (Murphy and Johnson, 2011). Biographies and life stories have been studied to explain the important role of childhood memories in the development process of leaders (Shamir et al., 2005), their values, and their behaviors. A leader’s personal history (such as family influences or early life challenges) and key trigger events (such as dramatic episodes) are antecedents in leadership development (Gardner et al., 2005) that enable personal development and that can support the development of certain qualities such as drive and determination (Turner and Mavin, 2008). Leader values and behaviors develop depending on how the individual internalizes or makes meaning of these formative experiences (Kayes, 2002; Sparrowe, 2005; Jefferson et al., 2014).

Leader values are beliefs that motivate behaviors and decisions. They are developed in a social context through culture, communities, and families. Good leaders integrate key values such as honesty, justice, and fairness, which support the process of decision making that have an effect on organizations (Russell, 2001). The role of values is essential in the development of leaders and leadership because values injected by caregivers can shape behaviors and attitudes (Russell and Stone, 2002). Family or community experiences related to collective trauma are influential in the development of a leader’s values because they can affect perceptions and behaviors (Meglino and Ravlin, 1998). According to researchers, exposure to traumatic events or stories of trauma, such as collective trauma, can instill values (Peterson and Seligman, 2003; Park and Peterson, 2009). Understanding the origin of these values is important when understanding leaders and leadership (Avolio and Gardner, 2005).

Narratives or memories relevant to collective traumas are considered as trigger events or stories that are remembered or stored (as repositories) in the memory through family narratives, community events or rituals (Kimbles, 2006), as we examine in the next section. A collective trauma, such as genocide or war, that may not have been personally experienced but shared in families or collective groups may not be known to the individual but still have an impact on the self. Collective trauma-related memories that may or may not have been spoken by family members may be unconsciously transmitted to the next generation (Fewell, 2016).

## Repositories of Collective Trauma

Research in psychology and cognitive psychology suggests that collective trauma resides in three sociocultural repositories: the cultural, communal, and familial. Within the context of collective trauma, families and communities can carry and transfer beliefs and values to their offspring that establish inherited memories or, as we identify, repositories (Danieli, 1998). Cultural structures including family and community (through stories, music, language, memorial events) influence how the collectiveness is stored and copied among descendants (Kupelian et al., 1998; Rousseau and Drapeau, 1998). In this section, we describe three key collective repositories of collective trauma that play a key role in the transmission process to descendants, namely, cultural rituals and artifacts, community events and commemorations, and family narratives.

### Cultural Rituals and Artifacts

Cultural artifacts and traditional rituals support collective memory and help develop self-identity (Assmann and Czaplicka, 1995; Jacobs, 2011). With the use of artifacts and rituals such as cultural, linguistic, or religious customs, individuals are able to relive the past, connect with the deceased, activate a belonging to a cultural group, and confirm their social identity (Beristain et al., 2000; Giladi and Bell, 2013). Artifacts and rituals enable individuals to connect with their historical trauma and their past and help group members sustain a social identity and connection with their culture. These artifacts and rituals give way to associations of feelings (i.e., anger, sadness) connected with the collective trauma. These feelings, in turn, can play a role in the development of the descendant’s identity (Hirsch, 2008; Sagi-Schwartz et al., 2008). Social learning (and in some cases social identity) is a mechanism by which cultural rituals or artifacts pertaining to a group are transmitted to the individual.

Giladi and Bell state that rituals and artifacts may promote determination, increased empathy, or the inclination to choose a profession that helps people, resilience, and a deep need to pass on the legacy of the trauma (Giladi and Bell, 2013). Cultural rituals and artifacts intensify the emotions shared by a collective group, giving them a sense of solidarity and an increased validation of their loss. Halbwachs (1992) and Beristain et al. (2000) deduce that rituals enable individuals to confirm their social identities by reconfirming the emotions that stem from collective traumas, thus enhancing self-esteem and social integration (Assmann and Czaplicka, 1995; Hirst and Manier, 2008).

Leaders who are third-generation descendants of collective trauma may not manifest symptoms of pathology, but they may be affected by the trauma that their forefathers and their ethnic group survived through the memories transmitted by cultural, linguistic, or religious mechanisms. The transmission of collective trauma is then processed through artifacts or rituals to communities, and eventually families; these artifacts or rituals help individuals and families maintain a connection with the group or community (Bar-On et al., 1998; Wiseman et al., 2006). We therefore propose the following:

*Proposition 1. Memories and meanings of collective trauma reside in cultural rituals and artifacts*.

### Community Events and Commemorations

Members of a community who share emotions refer to the term *collective emotion* (Barsade, 2002; Barsade and Gibson, 2012). Members of the group experience the same emotions, which also describe the cohesion of that group. Research in group processes and interpersonal behavior has shown that simply being a member of a group can cause an individual to experience the effects of group-based emotions (von Scheve and Ismer, 2013).

Communities and groups that have endured collective trauma are shaped by their group’s historical stories, cultural events, and memorial ceremonies (Schiff et al., 2001). These stories and events develop collective memories (Assmann and Czaplicka, 1995) to explain the representations and images with which a group associates itself (Hirst and Manier, 2008; Zembylas and Bekerman, 2015). These events may not be part of the cultural identity as explained in the previous section but instead may activate a story relevant to the individual’s history. Some researchers call this not only collective memory but also cultural memory, as the members of the group collectively feel that their identity and culture have been threatened. This threat leaves a mark on their collective consciousness, which also helps community members develop their collective identity (Schiff et al., 2001; Wohl et al., 2006; Hirst and Echterhoff, 2008; Ricoeur, 2009; Wohl and Branscombe, 2009).

According to Volkan (2001), a community or a group has a “mental representation” of its historical past, and a group that has endured a historical collective trauma shares losses and the emotions of mourning. Even if the individuals in that group have their own individual mechanisms of reacting to the collective trauma, they all share the mental representation of the trauma. Collective groups hold commemorations and memorials, which reactivate the emotions and anxieties of group members related to their perpetrators. Collective trauma, an event that may have taken place years or even a century ago, may feel as though it took place only recently, and this sense of recency also forms part of the group’s collective identity (Volkan, 2001; Fromm, 2012).

Communities actively attempt to maintain their culture, language, and religion to ensure that the legacy of their ancestors survives, fomenting a sense of retribution toward the aggressor state (Bezo and Maggi, 2015). Collective memory is relived through commemorations and rituals (Halbwachs, 1992), allowing individuals to maintain an identity as part of the group (Halbwachs, 1992; Assmann and Czaplicka, 1995; Paez et al., 2015).

Leaders who are members of a collective group develop their social identities through group membership (Schiff et al., 2001; Hirst and Echterhoff, 2008; Ricoeur, 2009; Wohl and Branscombe, 2009) because communities provide historical images or recollections of the collective trauma. Those images or recollections impact community and family dynamics, which then impact the leader as an individual member of that community or family (Ross, 2001). We therefore propose the following:

*Proposition 2. Memories and meanings of collective trauma reside in community events and commemorations*.

### Family Narratives (Said and Unsaid)

Memory transmitted by family members helps orient the individual; it is memory that helps develop the identity of the leader (Eyerman, 2004). Stories told over time (or the story communicated through silence) are represented, communicated, and expressed through various mechanisms (Wohl and Branscombe, 2009; Vollhardt and Bilewicz, 2013) such as through stories told, mournings, traditional songs, and books or movies. The family carries (consciously and unconsciously) the values, myths, and beliefs to the next generation, thus impacting the identity of the descendant.

Shared images or narratives of a collective traumatic event, such as genocide or war, can be transferred to descendants who, in turn, process this mourning (Volkan, 2009). Danieli suggests that trauma is transmitted as a family legacy regardless of whether family members talk about the event (Danieli, 1998). He identifies several effects or “themes” in second- and third-generation Holocaust descendants such as increased responsiveness to, and identification with, the Holocaust as experienced by their parents and grandparents. Kupelian also examines transgenerational issues among Armenian descendants of the 1915 genocide and finds that members of the third generations, who have heard stories from their grandparents about the injustice and how this genocide has yet to be acknowledged by the Turkish state, are overwhelmed with pain for the suffering of their forefathers (Kupelian et al., 1998; Bilali, 2013).

Many collective trauma survivors have refrained from talking about the events they witnessed during the genocide; they have expressed heavy emotions or sadness that may not even have been verbalized but that were nevertheless explicitly felt by their children (second generation), making the latter sensitive to their parents and their survival stories, told or untold (Bar-On et al., 1998; Wiseman et al., 2002). Even when the family does not communicate about the past, the results of the transmission of trauma can still be evidenced through emotions that are unconsciously displaced to the next generations (Dalgaard and Montgomery, 2015).

The leader who is a third- or fourth-generation descendant of collective trauma may not have lived through the trauma directly, but may be able to process emotions that have been transferred to them through the verbal or nonverbal recounting of narratives. This leader is likely to have noticed or felt the pain of their parents and grandparents, whose healing processes may have been ongoing. The values and even behaviors lived by their forefathers are transmitted to these leaders (Sanchez-Burks and Huy, 2009). We therefore propose the following:

*Proposition 3. Memories and meanings of collective trauma reside in family narratives*.

Having identified three nonexhaustive repositories of collective trauma that are capitalized in the acculturation of descendants, even from a young age, in the next section we describe the channels through which these collective trauma repositories are transmitted transgenerationally.

## Transgenerational Transmission of Collective Trauma

According to the psychoanalytical approach of Volkan (1998), victims of a large-scale collective trauma undergo several emotional psychodynamic processes, one of which is the transgenerational transmission of trauma. This large-scale trauma affects a group of individuals who share an affiliation (a national, cultural, or religious identity). Volkan identifies large-group identity as existing among a large group of people, many of whom may never know each other but most of whom share the same feeling. This “chosen trauma” is a mental reflection of the atrocities that the group’s forefathers endured (Volkan, 1998).

Research on this topic continues to grow and now includes studies of the grandchildren of trauma survivors (third generation) (Bar-On et al., 1998; Chaitin, 2002; Coles, 2011). The process of the transmission of trauma implies that the conscious and/or unconscious effects of trauma are transferred to children of survivors who have not witnessed the trauma. Collective trauma, more specifically, is transmitted to descendants through a communication process (through narratives, direct or silent) (Bar-On et al., 1998; Yehuda et al., 1998; Lev-Wiesel, 2007).

Volkan uses the depositing to explain how children are the primary active actors in collecting images and tasks from their environment and making these images and tasks their own. This transmission can affect the development of the child’s identity and thus their behavior (Volkan, 2001, 2009). When compared with second-generation survivors, third-generation descendants identify with their grandparents (the trauma survivors) more so than do second generations, although third generations show higher levels of psychological health and happiness (Lev-Wiesel and Amir, 2000; Lev-Wiesel, 2007). Some studies suggest that because grandchildren are more self-secure, they can allow themselves to identify with their grandparents. It has been argued that Holocaust survivors are better able to communicate their trauma experiences to their grandchildren toward the end of their lives, when they feel a greater need to communicate and leave their legacy behind (Danieli, 1998), and this communication facilitates the identification between grandparents and grandchildren.

Although prior research suggests that any individual who is a descendant can be affected by trauma, we focus our attention on how leaders may be developed as a result of the transmission process and how this transmission can affect their leadership values and behavior. A process of sensemaking related to a leader’s collective past can help “engage them to understand the ‘why’” of their leadership behavior, and subsequently help “bring meaning to the values and beliefs that underlie their behaviors in organizations” (Ostroff et al., 2013, p. 644).

To better understand the channels of transmission of collective trauma, we utilize three theories that can help us understand how collective historical trauma can be internalized–*social learning*, *social identity*, and *psychodynamics*–all of which help us understand the conscious and unconscious processes that can shape values of descendants. Figure 1 provides a heuristic model to help locate the transmission of collective trauma in a conceptual framework across cultural, community, and family levels of analysis.

### Transmission Through Social Learning

Social learning theory (more recently labeled social cognitive theory), as developed by Bandura, explains how people learn from each other through observation, imitation, and modeling (Bandura, 1969, 1989). According to this theory, observational learning begins during childhood as the child imitates her caregivers (Grusec, 1992). Based on this childhood learning, the behavior of an eventual leader is processed and impacted (Miller and Morris, 2016), as the leader learns by copying the values and behaviors of their models. This theory helps shed light on how situational influences such as role models can influence the characteristics of leaders (Brown and Treviño, 2006).

The social learning approach is grounded in the idea that behaviors are developed based on observations made in social contexts. As understood by Keller, leaders assume the values that they have observed in family settings and social environments (Keller, 2003). As a basis of this theory, we can assume that the emotions and narratives of collective trauma transmitted to children and grandchildren can facilitate the learning of emotions and the development of similar behaviors; eventually, these emotions and narratives should be identifiable in the values of descendants.

We propose that through the social learning channel, a collective group’s cultural rituals and artifacts reinforce the social-cultural influence on the members of the group by activating the transmission of the collective trauma through the observance or practice of rituals or use of the collective’s cultural artifacts (Beristain et al., 2000; Jacobs, 2011). Our proposition suggests the following:

*Proposition 4. When it concerns group’s cultural rituals and artifacts, collective traumas are transmitted through a social learning channel*.

### Transmission Through Social Identity

To understand whether leaders associated with a collective trauma implicitly or explicitly associate themselves with their collective group, we use social identity theory (Hogg, 2001) because it helps give meaning to this phenomenon. Leaders who are members of a collective or who share a collective historical trauma are presumed to have distinct characteristics that are connected to that group. According to social identity theory, a leader’s membership in a group shapes their attitudes, feelings, and behaviors (Ashforth and Mael, 1989), and these attributes mirror the group’s essence (van Knippenberg and Hogg, 2003). According to Tajfel and Turner (1979), history plays an important role in the development or formation of identity. Collective historical narratives, through commemorations and events, help provide a national identity and social cohesion (Korostelina, 2015). When leaders identify themselves with a group of people, they associate themselves with the characteristics of that group, and this association reinforces the antecedents of identification (Ashforth and Mael, 1989).

Intergroup emotions and behaviors can surface when leaders feel that a salient part of their identity is connected to membership in a group; this feeling leads to group-based emotions even if the individual is not an active member of the group (Tajfel and Turner, 1979). A leader’s identity with an ethnic group or with a collective history can predict leader values and behaviors (Johnson et al., 2012a).

We propose that through the social identity channel, community events, or ceremonies that relive or reassert the group’s collective trauma allow descendants to identify themselves with the legacy of that group, which helps sustain and reinforce values and behaviors that reflect their cultural heritage (Cox et al., 1991; Phinney et al., 2000). Therefore, we propose the following:

*Proposition 5. When it concerns community events or ceremonies, collective traumas are transmitted through a social identity channel*.

### Transmission Through Psychodynamics

The key foundation of psychodynamic theory is that all humans develop emotional and unconscious processes during childhood, and these processes help in the development of emotions, personalities, and behaviors (McLeod and Kettner-Polley, 2016).

The systems psychodynamic approach, which is linked to psychodynamic theory, explains that a child’s relationship with caregivers and home environments may consciously and unconsciously influence traits and qualities that are developed in their adult life and that are not clearly identified by the child’s consciousness (Kets de Vries, 2011, 2014). The “inner theatre” is a concept within the psychodynamic approach that explains how the people encountered in childhood influence emotions and experiences in the leader’s later life; these emotions and experiences, in turn, influence behavioral patterns (Neumann and Hirschhorn, 1999; Kets de Vries, 2003; Kets de Vries et al., 2013). Psychodynamic theory help us understand that leaders may incorporate behavioral patterns developed through psychological imprints from their early life and that caregivers may have affected the child’s social development (Marquis and Tilcsik, 2013).

Volkan uses psychodynamic theory to assess more closely how a sense of commonality and shared understanding is developed when an individual is part of a large group (national, religious or ethnic) (Volkan, 2001). When an individual is part of a large group (national, religious, or ethnic), there is a sense of commonality and shared understanding. Numerous studies show how individual responses to trauma result in anxiety and expectations that are passed on from parent to child, affecting the child’s sense of self and identity (Bar-On et al., 1998; Lev-Wiesel, 2007; Coles, 2011). To this effect, the leader who is part of an ethnic group that has suffered a collective trauma may have absorbed sensitivities, emotions, and characteristics modeled by their caregivers and resulting from stories told or untold, or traditions carried on, in response to the trauma.

We propose that through the psychodynamic channel, leaders who are descendants of a collective trauma absorb the values lived, felt, or told through stories told by their caregivers or collective family members (Crawford, 2014). We summarize this in the following proposition:

*Proposition 6. When it concerns family narratives, collective traumas are transmitted through a psychodynamic channel*.

Drawing on theories such as social learning, social identity, and psychodynamics, we suggest that the transgenerational transmission of collective trauma can take place through three processes: cultural rituals and artifacts, community events, and family narratives. All three of these processes are important mechanisms through which collective traumas are transmitted, influencing who leaders become, and how their cognition and behaviors are shaped. In the next section, we identify several values influenced by collective trauma that can shape leader cognition and behaviors, and which can impact how an organization is led. These are also the values (and possibly behaviors) that must be further researched if we are to understand the implications of the transmission of collective trauma on leader development, namely, leader values.

## Leader Values Stemming from the Transmission of Collective Trauma

Leader values can be processed through the information (knowledge) obtained and recalled by childhood experiences (Mumford et al., 2015). In our preliminary research on leaders who are descendants of the Armenian genocide, we have begun to notice evidence of how this collective trauma may have influenced third-generation leaders to develop values of perseverance, for instance, as a mechanism of resisting the victim mentality. In parallel, we identify research proposing that specific values and characteristics are triggered by the transmission of collective trauma and emerge during challenging incidents (Palgi et al., 2015). These values, such as resiliency, have been identified in the grandchildren of collective trauma survivors (Kellermann, 2001a,b; Scharf, 2007; Yehuda et al., 2008; Yehuda and Bierer, 2009; Letzter-Pouw et al., 2014). Wohl and Van Bevel substantiate that the psychological well-being of the descendant depends on how she interacts or associates herself with the collective group (Wohl and Van Bavel, 2011).

Collective trauma is still traumatic for third-generation leaders; however, successful leaders can take rich material from their history and transform it into something positive, productive and results driven, as anticipated by Peterson and Seligman (2003, 2004). In this section, we identify five values relevant to the transmission of collective trauma to leader descendants: resilience, forgiveness, empathy, justice, and perseverance.

### Resilience

Resilience is the characteristic that allows individuals to sustain, change, or modify their responses based on stressful events or situations (Bonanno, 2004; Tugade and Fredrickson, 2004). The effect of resilience is a common theme in the literature discussing the children and grandchildren of Holocaust survivors (Baron et al., 1996), who come from families with more open communication styles and in which the child-parent dynamic is more relaxed. Braga et al. demonstrate that national and cultural values and collective connections relevant to the collective trauma lead children and grandchildren of Holocaust survivors to develop resilience, particularly when they are faced with the need to defend or safeguard their nation, religion, and/or culture (Braga et al., 2012). In other words, trauma-related stories that are told to, or memories felt by, descendants of the collective trauma allow them to attain “inherited lessons” from their families and community members (Branscombe et al., 2015).

The transgenerational transmission of trauma does not necessarily signify a negative or pathological effect (van IJzendoorn et al., 2003; Levav et al., 2007; Sagi-Schwartz et al., 2008), but it can stimulate resilience and a need to pass on the legacy of the trauma. In other words, even if there is no pathology or negative effect, the trauma is still transmitted. Something is passed on to the next generation, and it can be in the form of posttraumatic growth and resilience (Giladi and Bell, 2013). Psychodynamic and social learning theories help us understand that third-generation survivors understand that there is emotional difficulty in addressing a traumatic past. This understanding has made it easier for third generation survivors to accept difficult personalities in the family, helped them appreciate strong family ties, and given them mechanisms with which to handle stressful situations (Kellermann, 2008).

Resilient leaders are considered effective at addressing change and innovation challenges in today’s world (Lane et al., 2013; Matzenberger, 2013) because they are able to rebound from negative situations and can adapt to changing or stressful demands (Tugade and Fredrickson, 2004). Individual and organizational behavioral perspectives can help explain why leaders are resilient and whether leader resilience is triggered by specific organizational actors or organizational contexts that increase resilience in leaders, who are descendants of collective trauma. We therefore propose the following:

*Proposition 7. Collective trauma transmission positively influences leaders’ resilience*.

### Forgiveness

Forgiveness (and its opposite, vengefulness) is a highly researched topic. Forgiveness is relevant when building professional relationships based on trust, but when leaders have vengeful emotions due to their traumatic past, their ability to exercise authentic or exemplary leadership may be hindered.

In attempting to overcome sad or damaging emotions triggered by the trauma experienced by family members, forgiveness helps to restore peace and create a culture of empathy (van Dierendonck and Patterson, 2015). When third-generation leaders have lived or felt the pain of surviving collective trauma from their parents and grandparents through stories told, memorial events, or cultural rituals, there is a need to feel that forgiveness heals painful memories and lead to inner peace.

Forgiveness may be an almost impossible action for descendants of collective groups whose trauma has not been acknowledged. The pain that lingers on from the trauma of forefathers–and the lack of acknowledgement of the trauma by the perpetrators–hinders the process of finding peace and cultivates vengefulness (Staub et al., 2005), and this can impact how a descendant of collective trauma performs as a leader or executive. This value–forgiveness–draws our attention to differences among leaders. A particular concern highlighted in this article is that a significant body of research on leader forgiveness rests on untested assumptions about the ethical behavior of leaders–assumptions related to different forgiveness behaviors exhibited by leaders.

In the authentic transformational leadership and ethical leadership literature, there is evidence that leaders demonstrate values such as forgiveness due to their grounded connection to family and community, and in relation to their cultural beliefs (Bass and Steidlmeier, 1999; Caldwell and Dixon, 2009; Fry and Kriger, 2009). We therefore propose the following:

*Proposition 8. Collective trauma transmission positively influences leaders’ forgiveness*.

### Empathy

Empathy is the ability to genuinely feel and understand the emotion and experience of another (Johnson et al., 2012a). While it may be difficult for victims of collective trauma to be empathetic given the physical, mental, and emotional pain they have endured, descendants of collective trauma may–based on the testimonies or narratives of their victim ancestors–have the capacity to be empathetic to others who have endured similar hardships. In many cases, and when using the social identity lens, empathy is developed when narratives of collective trauma include examples of kindness and goodness perpetuated by out-group members, or members of the persecuting group who helped victims escape the trauma, for instance.

Scholars have assessed how individual trauma increases empathetic behavior. Frazier assesses how exposure to trauma leads to increased spirituality and empathy for others who have been in similar situations (Frazier et al., 2013). Zembylas explains the process of transforming trauma narratives of the past (i.e., told by survivors or descendants of survivors through narratives or commemorative rituals) into values of empathy as a way for victims to come to terms with emotionally painful events (Zembylas, 2007).

Empathetic behaviors are evident when individuals are sensitive to circumstances to which they can relate, such as discrimination or harassment (Sosik and Megerian, 1999). According to social identity and social learning theories, leaders who have collective identities may feel the need to help members of a group or a collective by ensuring that the group benefits from their leadership role. Group or collective empathetic behaviors support leader members of the group to model similar behaviors (Johnson et al., 2012a). We therefore propose the following:

*Proposition 9. Collective trauma transmission positively influences leaders’ empathy*.

### Justice

Leaders hold positions from which they exert power in their organizations, and they have the opportunity to establish an environment that is fair and just. Because family and collective historical narratives, such as collective trauma, can shape and develop an individual’s identity (social identity), which then provides a clearer sense of self-knowledge and integrity. For individuals who are members of a collective group, collective stories provide the justification to lead with a sense of purpose (Shamir et al., 2005). Drawing on previous research (Avolio et al., 2004; Shamir et al., 2005; Day and Harrison, 2007), we can speculate that collective stories of trauma can both lead to greater self-clarity and purpose and become a source of internal guidance to individuals in their roles as just leaders.

When individuals share a collective history or a collective trauma, they can be collectively motivated by a sense of justice that connects them to their memory of their ancestors. A rich sociocultural family or community environment can nourish descendants with the sense of self (Sparrowe, 2005) and a connection with a sense of justice as minorities in their adopted countries or communities.

Leaders whose values have been transmitted by family narratives, community events, or cultural factors may use their collective trauma as a *moral compass* (Schwarz, 2000; Turner and Mavin, 2008). We therefore propose the following:

*Proposition 10. Collective trauma transmission positively influences leaders’ justice*.

### Perseverance

Research on authentic leadership has shown that personal history (family influences, role models, and early life challenges) and trigger events influence leaders (Gardner et al., 2005). Collective trauma and the narratives heard about that trauma in family and community settings may impel leader descendants to actively demonstrate perseverance. Grandchildren of collective trauma survivors, in contrast to their parents, try to ensure that they live successful lives to prove that they have excelled despite their past trauma. According to social learning and psychodynamic theories, third- and fourth-generations, having seen how their parents attempted to rebuild their lives, attempt to help sustain the legacy of their forefathers and safeguard their professional success (Bar-On et al., 1998). Prior research has identified antecedents and trigger events that can support the individual’s personal growth and development, suggesting that leaders can develop characteristics such as determination or perseverance (Avolio and Gardner, 2005; Shamir and Eilam, 2005; Turner and Mavin, 2008).

Values of perseverance may be developed due to a sense of purpose and a need to triumph over the pain or failure that an individual has seen through their caregivers (psychodynamically). The leader who has grown up in a family or community setting that has a background of collective trauma may have heard about a father or a grandmother who lost their livelihood and had to work hard to rebuild their life and sustain a cultural or ethnic legacy that was almost lost, which may explain or justify the development of hardworking and self-determined behavior in that leader.

In summary, values help provide the leader with a sense of purpose and generate behaviors that are relevant to leadership (Meglino and Ravlin, 1998; Lord and Brown, 2001). Leaders who have been exposed to family or community-related narratives, events or rituals may have developed values and behaviors that have been transmitted to them by their forefathers who were exposed to a collective trauma such as genocide. Further research is required to confirm what is transmitted to leader descendants and how stories, memorials and artifacts may have implicitly or explicitly shaped their values and behaviors. A potential subject for research in organizational theory and leadership theory is to assess how collective trauma helps develop leadership talent that supports organizational performance and impacts organizational behavior (Day, 2001; Solansky, 2015). We therefore propose the following:

*Proposition 11. Collective trauma transmission positively influences leaders’ perseverance*.

## Discussion and Future Research

The purpose of this article was to explore how the transmission of collective trauma shapes leader values. We addressed this by underlining the importance of collective trauma and leader values as constructs. We then identified the collective trauma repositories that are key in the memory of descendants as well as the channels through which these repositories are transmitted. We finally proposed a model (Figure 1) that explains how these repositories can shape leader values such as resilience, forgiveness, empathy, justice and perseverance, substantiated with relevant propositions.

By drawing on the case of the transmission of genocide as a collective trauma, our article makes three key contributions. First, we provide a deeper understanding of the transmission process and its role in the development of leader values. Next, we explore the relevance of three key theories in the transmission process (social learning, social identity, and psychodynamic). Finally, we shed new light on the consequences of collective trauma on the development of leaders for future contributions to the studies of leadership, organizational management, and organizational behavior.

The role of sociocultural influences on leader development has been important in the leadership development literature. Situational influences such as family, education, and the social environment have been assessed to explain why leaders behave the way they do (Shamir et al., 2005; Day et al., 2014; Mumford et al., 2015). Various studies have been developed to show that leadership cognition and behavior are not only influenced genetically but also shaped by society and the social context (Johnson et al., 2012b; Showunmi et al., 2016). There are also numerous studies relevant to how leaders got where they are today as a result of their psychodynamic life patterns (i.e., childhood imprints, sociocultural influences) (van de Loo, 2000; de Jager et al., 2003; Avolio and Gardner, 2005; Marquis and Tilcsik, 2013; Liborius, 2014). One topic that remains omitted, however, is the effects of collective traumas on leader development, specifically on leader values and behaviors.

The role of values is essential in the development of leaders and leadership because values injected by caregivers and community members can shape behaviors and attitudes (Russell and Stone, 2002). Family community and cultural experiences related to collective trauma are influential in the development of a leader’s values because they can affect perceptions and behaviors (Meglino and Ravlin, 1998). According to researchers, exposure to traumatic events or stories of trauma, such as collective trauma, can instill values (Peterson and Seligman, 2003; Park and Peterson, 2009).

There is room for future research to identify what, precisely, is transgenerationally transmitted by collective trauma and how this implicitly shapes descendants who are executives and leaders. It is also essential to identify triggers and stressors that are activated in the development of these leaders’ values. A deeper dive into the process by which collective trauma is transmitted would help us understand how this transmission process affects leaders and whether the values instilled in them as survivors of collective trauma has shaped them in their roles as leaders.

One conceptual problem that we have not addressed is the specific delineation between the collective trauma repositories and the relational values that may be activated. How do collective traumas specifically foster resiliency in leaders and how does this influence decision-making, followership and organizational development? An assessment of how leaders who are descendants of collective trauma may be more apt to display resilience and foster confidence among organizational stakeholders is needed (Avolio and Gardner, 2005; Gardner et al., 2005). A leader-follower perspective can be used to examine leader relations and how they affect decision-making. Scholars interested in leader traits and behavioral theories may also be interested in assessing–by analyzing leaders at the individual, group and organizational levels–whether leaders who have a connection to a historical collective trauma are more effective. From this perspective, future research could also explore how collective trauma produces ethical behavior in leaders who are descendants and how this can help followers emulate ethical conduct, consistent with social learning and social identity theories (Bandura, 1989; Treviño et al., 2000; van Knippenberg et al., 2004).

Researchers of intragroup and interpersonal behaviors may be interested in assessing the role of collective trauma as a way of understanding leader justice, as descendants of trauma may be guided by the moral social justice of their in-group community (Platow et al., 1997). Future research could build on the conceptualization of ethical, authentic or servant leadership to assess how collective trauma can activate leaders’ morality or sense of justice and how this encourages or discourages normative behavior in organizations (Gardner et al., 2005; Eubanks et al., 2012; Mayer et al., 2012).

Similarly, further studies that examine how and under which circumstances the values and behaviors of leaders are actually developed or executed will enable researchers to develop more accurate expectations about leader behaviors and develop a basis for research measuring the differences between leaders who are descendants of collective trauma compared to those who do not have such a history. For instance, how do leaders with a history of collective trauma enact empathy? How do these leaders recognize and respond to changes in the emotional state of other organizational members (Sosik and Megerian, 1999)?

Developing a better sense of how collective traumas influence leaders who are the descendants of such traumas provides a great opportunity to fill a void in our understanding of the role of, and possibilities for, leaders in organizations. Such research will bring new breadth to our understanding of organizational behavior and decision-making processes. It will also help open the doors to a greater understanding of leadership development in which leader-follower and collaborative relationships are increasingly required to attain business results and outcomes by strengthening and shaping organizational culture and engagement.

## Conclusion

Organizations today are colored by a growing number of diverse leaders who are descendants of ethnic cultures, some of whom have pedigrees of a historical collective trauma. There is little literature that examines the implications of a person’s collective trauma on their leadership values. This article attempted to draw on research on the transgenerational transmission of collective trauma to address the question of how these historical traumas can shape leader values. We explored the sociocultural repositories relevant to collective traumas and the channels by which they link to five nonexhaustive leader values and developed a list of propositions to assess these transmission relationships. We conclude this article with a discussion of how our theoretical insights can guide future research if we are to better understand origins and seeds of leader values.

## Author Contributions

LT is the leading author of the article. She conducted this research as a part of her PhD research. SK, as the primary PhD supervisor, was fully involved in the design, development and completion of the article. EL and RL as PhD co-supervisors provided intellectual contribution and developed various parts of the article.

### Conflict of Interest Statement

The authors declare that the research was conducted in the absence of any commercial or financial relationships that could be construed as a potential conflict of interest.
